# Enhanced Brain Stroke Lesion Segmentation in MRI Using a 2.5D Transformer Backbone U-Net Model

**DOI:** 10.3390/brainsci15080778

**Published:** 2025-07-22

**Authors:** Mahsa Karimzadeh, Hadi Seyedarabi, Ata Jodeiri, Reza Afrouzian

**Affiliations:** 1 Faculty of Electrical and Computer Engineering, University of Tabriz, Tabriz 51666, Iran; mahsakarimzadeh@tabrizu.ac.ir; 2 Faculty of Advanced Medical Sciences, Tabriz University of Medical Sciences, Tabriz 51656, Iran; 3 Miyaneh Faculty of Engineering, University of Tabriz, Miyaneh 51666, Iran; afrouzian@tabrizu.ac.ir

**Keywords:** brain stroke lesions, MRI images, U-Net neural network, diagnostic tools

## Abstract

Background/Objectives: Accurate segmentation of brain stroke lesions from MRI images is a critical task in medical image analysis that is essential for timely diagnosis and treatment planning. Methods: This paper presents a novel approach for segmenting brain stroke lesions using a deep learning model based on the U-Net neural network architecture. We enhanced the traditional U-Net by integrating a transformer-based backbone, specifically the Mix Vision Transformer (MiT), and compared its performance against other commonly used backbones such as ResNet and EfficientNet. Additionally, we implemented a 2.5D method, which leverages 2D networks to process three-dimensional data slices, effectively balancing the rich spatial context of 3D methods and the simplicity of 2D methods. The 2.5D approach captures inter-slice dependencies, leading to improved lesion delineation without the computational complexity of full 3D models. Utilizing the 2015 ISLES dataset, which includes MRI images and corresponding lesion masks for 20 patients, we conducted our experiments with 4-fold cross-validation to ensure robustness and reliability. To evaluate the effectiveness of our method, we conducted comparative experiments with several state-of-the-art (SOTA) segmentation models, including CNN-based UNet, nnU-Net, TransUNet, and SwinUNet. Results: Our proposed model outperformed all competing methods in terms of Dice Coefficient and Intersection over Union (IoU), demonstrating its robustness and superiority. Our extensive experiments demonstrate that the proposed U-Net with the MiT Backbone, combined with 2.5D data preparation, achieves superior performance metrics, specifically achieving DICE and IoU scores of 0.8153 ± 0.0101 and 0.7835 ± 0.0079, respectively, outperforming other backbone configurations. Conclusions: These results indicate that the integration of transformer-based backbones and 2.5D techniques offers a significant advancement in the accurate segmentation of brain stroke lesions, paving the way for more reliable and efficient diagnostic tools in clinical settings.

## 1. Introduction

Stroke is a leading cause of death and long-term disability worldwide, with an increasing incidence that poses a significant public health challenge [1]. Accurate and timely diagnosis is crucial for effective stroke management, as early intervention can significantly improve patient outcomes [2]. Magnetic Resonance Imaging (MRI) is the preferred imaging modality for diagnosing stroke due to its high resolution and ability to differentiate between various brain tissues [3,4]. However, the accurate segmentation of stroke lesions within MRI images remains a complex task, particularly given the variability in lesion appearance and the limitations of existing image segmentation techniques [5,6]. Advanced methods are needed to improve the accuracy and efficiency of lesion segmentation, ultimately aiding in better clinical decision-making [7,8].

Liu et al. made a significant contribution to stroke lesion segmentation by developing MSDF-Net (Multi-Scale Deep Fusion Network), which integrates fine and coarse details from multiple MRI modalities, improving segmentation performance across various scales. This multi-modal approach demonstrated how combining features from different scales can enhance model precision. Earlier, Liu et al. utilized a convolutional neural network (CNN) for stroke lesion segmentation on multi-spectral MRI data, proving the ability of deep learning models to manage the variability in lesion appearances across different MRI sequences, establishing a reliable tool for clinical diagnosis [9].

Building on these approaches, Tomita et al. proposed a 3D residual convolutional neural network (3D ResNet) for post-stroke lesion segmentation, introducing residual connections to mitigate vanishing gradients commonly encountered in 3D medical imaging [10]. Their work highlighted the importance of deep architectures for volumetric data. Similarly, Yang et al. introduced CLCI-Net, which enhances lesion boundary delineation by incorporating cross-level feature fusion and context inference [11]. This method effectively combines global and local contexts, allowing for improved boundary detection in chronic stroke cases.

In another advancement, Qi et al. developed X-Net, which leverages long-range dependencies and depthwise separable convolutions to segment stroke lesions efficiently [12]. By capturing spatial dependencies over larger areas, X-Net achieves high accuracy with reduced computational overhead. Verma et al. further extended the field by integrating quantitative lesion assessment into automatic segmentation models, offering a comprehensive tool for clinical decision-making [13].

Clérigues et al. tackled the challenge of acute and subacute stroke lesion segmentation by developing a multi-modal MRI-based model that utilized FLAIR, T1, and T2 imaging [14]. Their fully convolutional network (FCN) approach also demonstrated efficacy in ischemic stroke lesion segmentation from CT perfusion images. Lastly, Soltanpour et al. achieved significant improvements in segmentation accuracy for ischemic stroke lesions using a deep neural network trained on CT perfusion maps, showing the power of large datasets in boosting model performance [15].

Current medical image segmentation techniques predominantly use either 2D or 3D convolutional neural networks (CNNs), each with inherent limitations [16]. While 2D models are computationally efficient, they often fail to capture the full spatial context, leading to inaccuracies in segmenting lesions that span multiple slices [17,18,19]. On the other hand, 3D models leverage spatial information across all dimensions but are computationally demanding, prone to overfitting, and challenging to apply to small datasets or images with anisotropic resolution [20,21]. Although U-Net architectures, particularly those with convolutional backbones like ResNet or EfficientNet, have improved segmentation accuracy, they struggle with 2.5D MRI data due to their inability to fully exploit inter-slice spatial dependencies and their substantial computational requirements.

In recent years, several advanced architectures have emerged for medical image segmentation. nnU-Net, proposed by Isensee et al. [22], is a self-configuring framework based on U-Net that automatically adapts its architecture and training pipeline to a given dataset and has achieved top performance in many segmentation challenges. TransUNet, introduced by Chen et al. [23], combines CNN-based feature extraction with Vision Transformers for long-range context modeling, providing a hybrid approach suitable for 2D medical segmentation. SwinUNet, proposed by Cao et al. [24], utilizes the Swin Transformer backbone with hierarchical attention to achieve effective local–global context integration, tailored for medical images. These models represent the current frontier in deep learning-based medical segmentation and are considered strong baselines for any novel approach.

To further situate our work within recent developments, we have expanded the literature review to incorporate both advances in deep learning-based segmentation and the emerging use of 2.5D strategies. Wang et al. proposed the MSTP-Net, a multi-scale three-path network for retinal vessel segmentation that effectively integrates multi-scale features and pathways, demonstrating strong segmentation performance and feature fusion techniques [25]. Closer to our application domain, Xue et al. developed a multi-path 2.5D convolutional neural network for automated stroke lesion segmentation using multi-modal MRI from ATLAS, indicating the feasibility and potential of 2.5D models in stroke imaging [26]. Furthermore, recent surveys such as Azad et al. underscore the growing importance of Vision Transformer-based architectures in medical image analysis [27]. Despite these advances, the combination of 2.5D spatial context with a lightweight MiT-based Transformer encoder for ischemic stroke MRI remains underexplored. Our work bridges this gap by introducing a novel hybrid architecture that balances inter-slice context, global attention, and computational efficiency, contributing new insights to the evolving field of efficient medical image segmentation.

**Figure 1:**
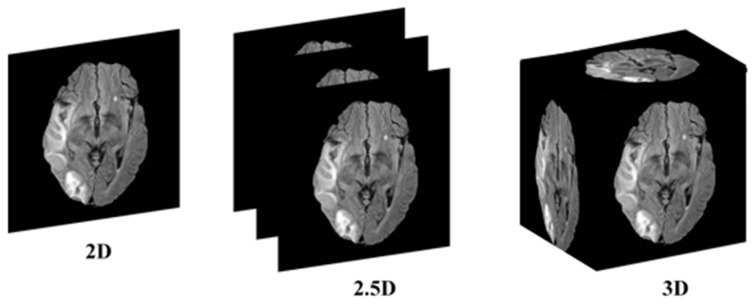
Conceptual illustration of 2D, 2.5D, and 3D segmentation inputs. In 2D segmentation, individual slices are processed independently, with no information shared across adjacent slices. In 2.5D segmentation, multiple adjacent slices (e.g., three) are stacked as input channels to capture inter-slice context, while retaining a 2D model architecture. In 3D segmentation, the entire MRI volume is processed using 3D convolutions, incorporating full volumetric context at the cost of higher computational demand.

This research addresses the mentioned gaps by integrating a transformer-based backbone, specifically the Mix Vision Transformer (MiT), into the U-Net architecture. The transformer’s ability to capture long-range dependencies and global context provides a more holistic understanding of the complex spatial relationships in 2.5D MRI images, enhancing stroke lesion segmentation accuracy while maintaining computational efficiency. The 2.5D picture segmentation problem is a significant, but little-studied, segmentation task. The goal is to segment stroke lesions using a 2.5D image representation, where spatial context across adjacent slices is incorporated (Figure 1).

The remainder of this paper is structured as follows: Section 2 details our proposed methodology, describing the U-Net model architecture with the MiT Backbone and the implementation of the 2.5D segmentation strategy. Section 3 presents our experimental setup, including a comparison of the transformer-based U-Net with ResNet and EfficientNet backbones, and discusses the results obtained from the 2015 ISLES dataset. Section 4 explores the implications of our findings for clinical practice and future research directions. Finally, Section 5 concludes this paper by summarizing the key contributions and proposing avenues for further investigation.

## 2. Methods

**Figure 2:**
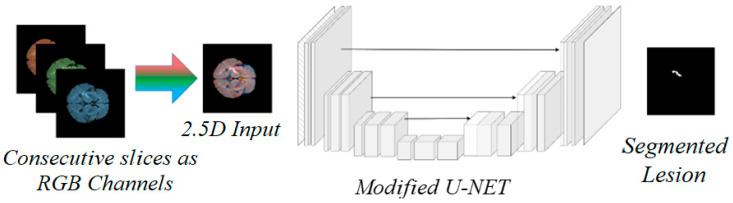
Conceptual diagram of the proposed 2.5D MiT-based U-Net model. The figure illustrates the general encoder–decoder structure with skip connections. Consecutive slices are processed as RGB channels to form 2.5D input, which is fed through the modified U-Net architecture to generate segmented lesion output.

In this work, we propose a novel framework for brain stroke lesion segmentation, combining the strengths of three key components: a 2.5D model, a Vision Transformer (ViT) backbone, and an encoder–decoder style network (Figure 2). The 2.5D model leverages consecutive MRI slices, capturing spatial context between adjacent slices without the computational complexity of fully 3D models. To enhance global feature extraction, we integrate the Vision Transformer, which excels at capturing long-range dependencies and global context across image patches. This is paired with a U-Net-based encoder–decoder architecture, where the encoder extracts multi-scale features and the decoder reconstructs the segmentation map with the aid of skip connections, preserving spatial details. Together, these components create a powerful segmentation model that balances computational efficiency with high accuracy, particularly for challenging medical image segmentation tasks like stroke lesion detection.

### 2.1. The 2.5D Model

Our 2.5D method processes MRI data by treating each scan as a sequence of 2D slices. For each target slice, we incorporate adjacent slices to enrich the spatial information available for segmentation. Specifically, for each input to the network, we stack three consecutive slices— the slice of interest (central slice), the preceding slice, and the following slice— along the channel dimension. This forms a multi-channel 2D input, where each channel corresponds to one of the slices. This technique allows the model to capture inter-slice information without the need for complex 3D convolutions.

The 2.5D approach significantly reduces computational overhead compared to 3D models, as it avoids processing entire volumes of data in a single pass. Instead, the model focuses on local regions with contextual support from adjacent slices, which is particularly useful for segmenting stroke lesions, where spatial continuity across slices is critical for accurate boundary detection.

One potential concern when using adjacent slices in a 2.5D setup is the possibility of slight misalignments across slices. However, since the data used in this study consists of volumetric 3D MR images, which are inherently spatially coherent due to the nature of the acquisition process, no explicit inter-slice alignment or registration was necessary. Instead, we applied standard preprocessing steps such as intensity normalization and bounding box cropping to ensure consistent spatial framing and intensity distribution. These steps help maintain anatomical consistency across slices without introducing artifacts from additional spatial transformations.

By utilizing the 2.5D method, our model benefits from the simplicity of 2D networks while still capturing some of the spatial dependencies found in 3D volumes. This balance between efficiency and accuracy makes the 2.5D approach an attractive choice for clinical applications where computational resources are limited but precision remains critical.

### 2.2. Vision Transformer (ViT)

In our approach, we incorporate a Vision Transformer (ViT) within the encoder to leverage its ability to capture long-range dependencies and global context, which are essential for accurate brain stroke lesion segmentation. Unlike traditional convolutional networks that rely on localized receptive fields, transformers can model relationships across the entire image. This global awareness is especially valuable for medical imaging tasks, where both local features and larger anatomical structures must be considered. To achieve this, we divide each MRI slice (or a stack of slices in the 2.5D method) into non-overlapping patches, which are then linearly embedded into a sequence of feature vectors. These vectors are further enriched with positional information to retain the spatial structure of the image.

The transformer encoder processes these patch embeddings using layers of multi-head self-attention and feedforward networks. This enables the model to attend to different regions of the image simultaneously, learning how distant parts of the brain might be interconnected, which is particularly useful in detecting subtle or dispersed stroke lesions. By focusing on both local and global features, the Vision Transformer provides a more comprehensive representation of the image, allowing the network to make more informed segmentation decisions. The resulting sequence of features is reshaped back into a 2D feature map, which is then passed to the decoder for further processing.

When integrated with the U-Net architecture, the ViT acts as a replacement or complement to the traditional convolutional encoder. This allows the model to maintain the U-Net’s strength in capturing fine-grained spatial details through skip connections, while benefiting from the ViT’s ability to model long-range relationships. The combination of convolutional layers for local feature extraction and transformer layers for global context leads to improved segmentation accuracy, particularly for complex lesions. This hybrid approach enhances the model’s ability to segment stroke lesions with high precision while remaining computationally efficient. It is notable that the encoder was initialized using publicly available pre-trained weights from ImageNet-1K, as part of the SegFormer [28] implementation.

### 2.3. Encoder–Decoder Style Network

To achieve accurate segmentation of brain stroke lesions from MRI images, we employ a modified U-Net architecture, which is a well-established convolutional neural network (CNN) designed specifically for medical image segmentation. The U-Net model is characterized by its symmetric encoder–decoder structure, where the encoder extracts features from the input image and the decoder reconstructs the segmentation map while maintaining spatial precision.

The encoder network is responsible for learning hierarchical feature representations from the input data. In our implementation, we replace the traditional U-Net encoder with a more powerful backbone to improve feature extraction capabilities. Specifically, we experiment with different backbones, including ResNet, EfficientNet, and the MiT, each of which introduces varying levels of abstraction and global context.

The encoder consists of multiple convolutional blocks, each followed by batch normalization and ReLU activation, ensuring stable and efficient training. Max pooling layers are employed after each block to progressively reduce the spatial dimensions of the input while increasing the depth of the feature maps, capturing both fine and coarse features. The final encoder layer outputs a compact yet informative feature representation of the input image. In the case of the MiT Backbone, the traditional convolutional layers are complemented by self-attention mechanisms, allowing the model to capture both local and global dependencies within the image. This provides an additional advantage, especially for complex structures like stroke lesions, which may require both detailed and contextual understanding for accurate segmentation.

The decoder network is tasked with upsampling the low-resolution feature maps produced by the encoder to the original input resolution, ultimately generating the segmentation mask. Our decoder mirrors the encoder’s architecture in reverse, using a series of upsampling layers (transposed convolutions) to progressively increase the spatial resolution of the feature maps. To ensure that important spatial details are preserved during upsampling, we incorporate skip connections between corresponding layers in the encoder and decoder. These skip connections directly transfer high-resolution features from the encoder to the decoder, helping the network recover fine details that may be lost during downsampling. This is particularly important for medical image segmentation, where precise boundary delineation is critical for accurate lesion identification.

Each decoder block consists of a transposed convolution layer, followed by a series of convolutional layers with batch normalization and ReLU activations. These operations refine the upsampled feature maps and recover spatial information from the encoder. The final layer of the decoder applies a 1 × 1 convolution followed by a sigmoid activation function, producing a probability map indicating the likelihood of each pixel belonging to the lesion class. During inference, we apply a fixed threshold of 0.5 to convert the probability map into a binary segmentation mask, which is used for performance evaluation.

### 2.4. Detailed Architecture Description

The input to the model is a 2D image that is 128 × 128 pixels in size. For 2D models, each input image has 1 channel. For 2.5D models, three consecutive axial slices are stacked along the channel dimension to form a 3-channel image. This allows the model to capture inter-slice dependencies in a way analogous to RGB images. The first convolution layer of the encoder is adapted to accept 3-channel inputs for the 2.5D variant; this modification does not significantly change the number of parameters.

The encoder consists of 4 MiT stages. Each stage includes a patch embedding layer (patch size 4 × 4), followed by a series of transformer blocks using multi-head self-attention and feed-forward layers. The number of blocks in each stage is {2, 2, 6, 2}, with corresponding embedding dimensions of {64, 128, 320, 512}. The spatial resolution is reduced at each stage by a factor of 2 through strided convolutions.

The decoder mirrors the encoder with 4 upsampling stages. Each stage uses transposed convolutions followed by two 3 × 3 convolutional layers with batch normalization and ReLU activation. Skip connections bridge the encoder and decoder at matching resolutions to preserve spatial information.

The final decoder layer applies a 1 × 1 convolution to produce a single-channel probability map, which is then passed through a sigmoid activation to generate the binary segmentation mask.

### 2.5. Loss Function and Class Imbalance Handling

Stroke lesion segmentation presents a typical case of class imbalance, as lesions usually occupy a much smaller region compared to the background. To address this, we used a hybrid loss function combining Dice Loss and Binary Cross-Entropy (BCE):

Loss = 0.7 × Dice Loss + 0.3 × BCE

Dice Loss promotes region-level overlap, which is critical for detecting small lesion regions, while BCE ensures stable pixel-wise convergence. This combination leverages the strengths of both losses: Dice’s robustness to imbalance and BCE’s ability to refine predictions in uncertain areas. All models were trained with this combined loss unless otherwise specified.

## 3. Experiments

### 3.1. Dataset and Preprocessing

In this study, we utilized the ISLES 2015 dataset from the SICAS Medical Image Repository, which contains multi-modal MRI scans of 20 individuals diagnosed with acute ischemic stroke. Among the available modalities, we selected the FLAIR (Fluid-Attenuated Inversion Recovery) sequences due to their high lesion-to-background contrast, which is particularly suitable for stroke lesion segmentation. Each subject contributes approximately 153 axial slices, including both original and lesion-specific annotations. For training, we randomly selected 16 patients to ensure a representative and robust sample [19].

The initial dataset consisted of 3D MRI scans with dimensions of 230 × 230 × 153 and a resolution of 1 × 1 × 1 mm^3^. To adapt the data for deep learning segmentation techniques, we preprocessed the images by converting the 3D MRI scans into 2D images along the axial plane. Non-informative images were excluded to optimize the dataset. Similarly, the ground truth data for ischemic stroke lesions underwent the same preprocessing procedure. During training, we calculated predictive accuracy using the prepared training data. During preprocessing, slices with no anatomical content (e.g., black or empty slices at the top and bottom of the volume) were excluded. However, all valid slices containing brain tissue—regardless of whether a lesion was present—were retained to ensure the model is trained on both lesion-present and lesion-absent examples. This step is critical to avoid overfitting and reduce false-positive predictions on healthy brain regions. Furthermore, we reduced the image size by trimming unnecessary regions and eliminating extraneous zero-padding during the up- and downsampling process.

The final image size was standardized to 128 × 128 × 3. To further improve image clarity, we applied Contrast Limited Adaptive Histogram Equalization (CLAHE), a technique that enhances specific sections of the image while maintaining consistency in high-contrast areas. CLAHE works by dividing the image into several non-overlapping sections, enhancing each part individually, and then merging the enhanced sections using interpolation, resulting in a more detailed and refined image.

### 3.2. Experimental Setup

We compared the performance of three popular backbone architectures integrated into a U-Net framework: ResNet, EfficientNet, and MiT. Each backbone was evaluated using two model configurations: 2D and 2.5D. The 2D models processed individual slices independently, while in the 2.5D configuration, three consecutive slices (the current slice along with its adjacent neighbors) were concatenated along the channel dimension, forming a three-channel input analogous to RGB images, balancing the spatial context of 3D models with the computational efficiency of 2D methods.

To validate the superiority of our model, we additionally implemented and benchmarked the following SOTA models under the same preprocessing and cross-validation scheme: nnU-Net (3D), TransUNet (2D), and SwinUNet (2D). The same 4-fold validation and image preparation pipeline was maintained to ensure fair comparison.

The experiments were conducted on a high-performance computing cluster with an NVIDIA RTX 4090 GPU(NVIDIA Corporation, Santa Clara, CA, USA), utilizing PyTorch version 2.1.0 (https://pytorch.org/) frameworks. We trained each model for 100 epochs, using a batch size of 32. To optimize the network weights, we employed the Adam optimizer with a learning rate of 0.001. To improve training stability, we implemented early stopping to prevent overfitting by halting the training if the validation loss did not improve for 7 consecutive epochs. Additionally, we used a learning rate scheduler to reduce the learning rate by a factor of 0.1 if the validation loss plateaued for 5 epochs. These strategies helped ensure better convergence and optimal performance across different models. Two-dimensional data augmentation techniques, including random rotations in the range of ±15 degrees, random scaling with factors between 0.9 and 1.1, random horizontal flipping with 50% probability, and random brightness/contrast adjustment, were applied to increase the variability of the training set and prevent overfitting.

These training strategies were consistently applied across all models to ensure fair comparison. We observed that the use of early stopping and learning rate scheduling particularly benefited the Transformer-based models (MiT), as they helped reduce overfitting and promoted smoother convergence. The MiT-U-Net exhibited more stable validation loss curves compared to CNN-based architectures, likely due to its stronger global context modeling, which was further enhanced by controlled training dynamics.

### 3.3. Evaluation Metrics

The performance of each model was assessed using three main evaluation metrics: Dice Coefficient (DICE), Intersection over Union (IoU), and Boundary F1 Score (BF1). The Dice Coefficient measures the overlap between predicted and ground truth segmentations, providing a direct indicator of segmentation accuracy, and IoU quantifies the extent of the intersection between the prediction and the ground truth, reflecting how well the model captures the lesion area. All evaluation metrics were calculated on binarized segmentation masks obtained by thresholding the model’s probability output at 0.5.

In addition to reporting the mean and standard deviation of three main evaluation metrics, we conducted paired Wilcoxon signed-rank tests to evaluate the statistical significance of the observed differences between models. This non-parametric test was chosen due to the limited sample size (4-fold cross-validation). Statistical significance was assessed at a threshold of *p* < 0.05.

### 3.4. Model Configuration

Before diving into the specific model configurations, it is important to highlight some key aspects of our experimental setup. We utilized pre-trained weights for the encoder components of all models to accelerate convergence and leverage learned feature representations. By initializing with these weights, the models could focus on learning domain-specific patterns more efficiently. Throughout the experiments, we explored both 2D and 2.5D approaches to examine how information from neighboring slices could be utilized, particularly in cases where the volumetric context is crucial. This allowed us to compare the performance of different architectures under varying input dimensions, providing insights into the strengths of each model design for medical image segmentation tasks. The implemented models are as follows:

• ResNet-Based U-Net:

The ResNet-based U-Net served as a baseline for our experiments. ResNet, known for its ability to handle deep networks using residual connections, was integrated into the U-Net encoder, enabling the model to learn hierarchical features while preserving gradient flow. Both 2D and 2.5D variants were trained, and the results were used as benchmarks for comparison.

• EfficientNet-Based U-Net:

EfficientNet is known for its compound scaling approach, which balances depth, width, and resolution to improve model performance while maintaining efficiency. We integrated EfficientNet into the U-Net encoder, hypothesizing that its efficient feature extraction would enhance segmentation accuracy, especially when combined with 2.5D data inputs.

• Mix Vision Transformer (MiT)-Based U-Net:

The core innovation of this research is the integration of the MiT into the U-Net architecture. MiT leverages self-attention mechanisms to capture long-range dependencies, providing a global context that is often lacking in traditional convolutional networks. The 2.5D configuration with MiT was designed to maximize the capture of inter-slice information, hypothesizing that this would lead to superior segmentation performance.

## 4. Results

The performance of the proposed U-Net models, integrated with different backbones, was evaluated on a segmentation task using both 2D and 2.5D configurations. The models included ResNet, EfficientNet, and MiT as the backbone networks. Each model was trained and tested using a high-performance computing cluster equipped with NVIDIA RTX 4090 GPUs, leveraging the PyTorch framework.

### 4.1. Quantitative Results

The performance metrics for all models, including DICE Coefficient, IoU, and BF1, are summarized in Table 1. The results present the mean and standard deviation obtained from a 4-fold cross-validation performed on the test data. Importantly, the number of trainable parameters was kept constant between the 2D and 2.5D variants of each model to ensure a fair comparison, isolating the impact of inter-slice context modeling.

As shown, our proposed 2.5D MiT U-Net model achieved the highest Dice score (0.8153), IoU (0.7835), and BF1 (0.863), demonstrating superior lesion delineation performance both in terms of region overlap and boundary accuracy. Compared to SwinUNet and TransUNet—both of which are transformer-based but limited to 2D inputs—our model benefited from enhanced inter-slice context while maintaining computational efficiency. Additionally, even the 3D nnU-Net, a strong baseline, was outperformed in all metrics despite its greater model complexity and memory demands. Statistical analysis (Wilcoxon signed-rank test, *p* < 0.05) confirmed that the 2.5D MiT U-Net significantly outperforms other backbone configurations in all three metrics

To validate the significance of performance improvements, we conducted Wilcoxon signed-rank tests on the Dice and IoU scores across all 4 folds. The results showed that the proposed MiT-based U-Net (2.5D) significantly outperforms the ResNet and EfficientNet-based U-Nets (2.5D) in both Dice and IoU metrics:

• MiT vs. ResNet (2.5D):
  ⚬ Dice: *p* = 0.028
  ⚬ IoU: *p* = 0.036
• MiT vs. EfficientNet (2.5D):
  ⚬ Dice: *p* = 0.042
  ⚬ IoU: *p* = 0.049

These findings confirm that the superior performance of the MiT-based U-Net (2.5D) is not only consistent but also statistically significant.

### 4.2. Performance Analysis

The MiT Backbone U-Net achieved the highest performance across all metrics in both 2D and 2.5D configurations. This superior performance can be attributed to the ability of MiT to capture long-range dependencies through self-attention mechanisms, providing a global context that traditional convolutional layers lack. This is especially beneficial in the context of medical image segmentation, where accurate delineation of structures can depend on understanding both local and global patterns.

Interestingly, the 2.5D variants of all backbones consistently outperformed their 2D counterparts. This suggests that utilizing additional inter-slice information helps capture more comprehensive spatial features, leading to improved segmentation accuracy. Notably, the MiT Backbone U-Net (2.5D) achieved the best DICE score (0.8153) and IoU (0.7835), demonstrating its ability to leverage the additional context provided by the 2.5D data.

Although the MiT Backbone provided the best results, it required longer training times before triggering early stopping. This can be explained by the more complex architecture of transformers, which takes additional epochs to converge compared to conventional convolutional networks. The benefits, however, outweigh the increased computation time, especially in scenarios where high accuracy is essential.

In addition to accuracy, we evaluated the training efficiency and hardware requirements of each model. On an NVIDIA RTX 4090 GPU, the MiT-based U-Net (2.5D) required ~3.7 h for training and ~17.8 GB of video memory, while the ResNet-based U-Net (2D) required ~2.1 h and ~11.2 GB. The 3D nnU-Net baseline required the longest training time (~5.4 h) and the highest GPU usage (~23.5 GB). Inference time per image was ~24 ms for our model, which supports real-time clinical deployment. These metrics demonstrate that our method is computationally efficient and scalable in real-world environments.

To evaluate generalization, we applied our trained 2.5D MiT U-Net model to the ISLES 2017 dataset without retraining. Despite differences in acquisition and annotation standards, our model achieved a Dice score of 0.7624 and an IoU score of 0.7211 on the ISLES2017 validation set, confirming the model’s ability to generalize beyond the original training data. Additionally, our analysis of slices without stroke lesions revealed a false positive rate below 2%, demonstrating the model’s reliability on normal brain regions and its applicability for full-volume inference.

### 4.3. Qualitative Results

**Figure 3:**
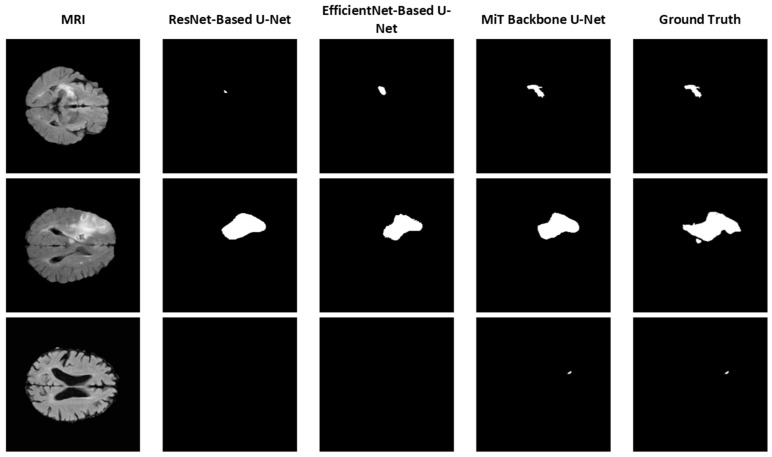
Sample segmentation results using different U-Net backbones (2D model).

To further illustrate the effectiveness of each model, segmentation results from the 2D versions of the ResNet-Based U-Net, EfficientNet-Based U-Net, and MiT Backbone U-Net are provided in Figure 3. For simplicity, we focus on comparing the backbones using 2D models to assess the impact of different backbones on segmentation performance. These examples highlight the varying levels of detail and segmentation accuracy achieved by the models. The MiT Backbone U-Net demonstrates the most precise boundary detection and delineation of structures, confirming the quantitative findings.

Figure 3 shows sample segmentation results comparing different U-Net architectures against ground truth annotations. For simplicity, we focus on comparing the backbones using 2D models to assess the impact of different backbones on segmentation performance. These examples highlight the varying levels of detail and segmentation accuracy achieved by the models. The MiT Backbone U-Net demonstrates the most precise boundary detection and delineation of structures, confirming the quantitative findings.

### 4.4. Training Dynamics

**Figure 4:**
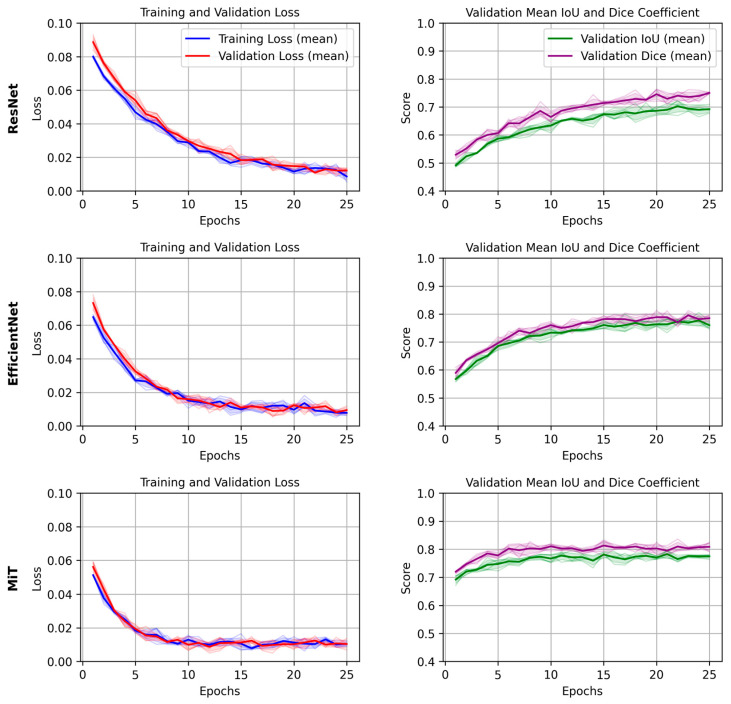
Mean and standard deviation of training and validation loss, and validation IoU/Dice scores across 4 folds for each U-Net backbone (ResNet, EfficientNet, and MiT). Shaded areas represent the standard deviation across folds, demonstrating the consistency and robustness of training and evaluation.

The training and validation loss curves, along with the DICE/IoU scores for each backbone (2D models), are plotted in Figure 4. For simplicity, we focus on the results from a single epoch to compare the models. The graphs provide insights into how quickly the models converged during training and how well they generalized to the validation set. The MiT Backbone U-Net shows a steady decrease in loss and a consistent increase in DICE/IoU during training, albeit at a slower rate compared to the other backbones. This behavior aligns with the longer training time needed for the model to reach optimal performance. In contrast, the ResNet and EfficientNet-based models showed quicker convergence, yet their final performance did not match that of the MiT Backbone U-Net.

## 5. Discussion and Conclusions

The results clearly demonstrate that the choice of backbone plays a significant role in segmentation performance. The MiT Backbone U-Net was the most effective, owing to its ability to integrate both local and global features through self-attention. While this architecture required more time to converge, it resulted in superior performance metrics, which is crucial in medical applications where accuracy is a top priority.

Additionally, the 2.5D configurations improved segmentation across all backbones, validating the hypothesis that incorporating inter-slice information can enhance model performance. These findings encourage further exploration of 2.5D and 3D data inputs, especially when using advanced architectures like transformers that can handle more complex data patterns.

The inclusion of SOTA models like nnU-Net, SwinUNet, and TransUNet in our comparative analysis confirms the robustness of our approach. Our model not only achieves the highest DICE and IoU scores but does so using a 2.5D approach that is computationally more efficient than full 3D models like nnU-Net. This has direct implications for clinical environments where GPU resources and inference time are limited.

In addition to the commonly used Dice and IoU metrics, which primarily assess region-level overlap, we also evaluated our model using the BF1 to better capture boundary-level segmentation performance. This metric is particularly relevant in stroke lesion segmentation, where precise delineation of lesion boundaries can influence treatment planning. Our analysis showed that the proposed 2.5D MiT-based model achieved a consistently higher BF1 score compared to other backbones, indicating improved accuracy in modeling lesion contours and fine structures.

Overall, the MiT Backbone U-Net stands out as the preferred model for this segmentation task, offering a balance between cutting-edge performance and the ability to generalize across variations in medical imaging data. Future work could explore optimizing the training process to reduce convergence time or integrating hybrid approaches that combine the efficiency of EfficientNet with the global context capabilities of MiT.

In summary, this study provides insights into the application of different backbones within U-Net architectures, highlighting the benefits of advanced models like MiT for achieving state-of-the-art segmentation performance in medical imaging.

## Figures and Tables

**Table 1:** Performance comparison of U-Net models with different backbones and input strategies on the test set. Metrics include Dice Coefficient, Intersection over Union (IoU), and Boundary F1 Score (BF1), reported as mean ± standard deviation over 4-fold cross-validation.

U-Net Backbones	#Params	DICE (Mean ± Std)	IoU (Mean ± Std)	BF1 (Mean ± Std)
ResNet-Based U-Net (2D)	11M	0.7490 ± 0.0112	0.7133 ± 0.0106	0.798 ± 0.0101
ResNet-Based U-Net (2.5D)	11M	0.7575 ± 0.0108	0.7180 ± 0.0095	0.806 ± 0.0097
EfficientNet-Based U-Net (2D)	10M	0.7959 ± 0.0103	0.7616 ± 0.0097	0.838 ± 0.0094
EfficientNet-Based U-Net (2.5D)	10M	0.8091 ± 0.0110	0.7744 ± 0.0089	0.851 ± 0.0087
TransUNet (2D)	14M	0.7861 ± 0.0131	0.7542 ± 0.0116	0.833 ± 0.0108
SwinUNet (2D)	15M	0.8012 ± 0.0105	0.7694 ± 0.0089	0.846 ± 0.0083
nnU-Net (3D)	~33M	0.7935 ± 0.0118	0.7669 ± 0.0094	0.841 ± 0.0092
MiT Backbone U-Net (2D)	13M	0.8015 ± 0.0095	0.7701 ± 0.0087	0.847 ± 0.0080
MiT Backbone U-Net (2.5D)	13M	**0.8153 ± 0.0101**	**0.7835 ± 0.0079**	**0.863 ± 0.0074**

## Data Availability

This study utilized publicly available datasets. Details regarding data sources and access are provided in the Methods section.

## Abbreviations

The following abbreviations are used in this manuscript:

MiT	Mix Vision Transformer
DICE	Dice Coefficient
IoU	Intersection over Union
CNN	Convolutional Neural Network
ResNet	Residual Convolutional Neural Network
ViT	Vision Transformer
MSDF-Net	Multi-Scale Deep Fusion Network
CLAHE	Contrast Limited Adaptive Histogram Equalization
